# The LRRK2–macroautophagy axis and its relevance to Parkinson's disease

**DOI:** 10.1042/BST20160265

**Published:** 2017-02-15

**Authors:** Claudia Manzoni

**Affiliations:** 1 School of Pharmacy, University of Reading, Whiteknights, Reading RG6 6AP, U.K.; 2 Department of Molecular Neuroscience, University College London, Queen Square House, London WC1N 3BG, U.K.

**Keywords:** autophagy, leucine-rich repeat kinase, neurodegeneration, Parkinson's disease

## Abstract

A wide variety of different functions and an impressive array of interactors have been associated with leucine-rich repeat kinase 2 (LRRK2) over the years. Here, I discuss the hypothesis that LRRK2 may be capable of interacting with different proteins at different times and places, therefore, controlling a plethora of diverse functions based on the different complexes formed. Among these, I will then focus on macroautophagy in the general context of the endolysosomal system. First, the relevance of autophagy in Parkinson's disease will be evaluated giving a brief overview of all the relevant Parkinson's disease genes; then, the association of LRRK2 with macroautophagy and the endolysosomal pathway will be analyzed based on the supporting literature.

## Introduction

Leucine-rich repeat kinase 2 (*LRRK2*) is the gene most frequently mutated in familial Parkinson's disease (PD); seven coding mutations characterized by population-specific penetrance (N1437H, R1441G, R1441C, R1441H, Y1699C, G2019S and I2020T) are cause of dominant, late-onset PD [1], while coding and noncoding polymorphisms in the LRRK2 locus are associated with an increased risk of sporadic disease [2]. Interestingly, PD is not the sole disease in which *LRRK2* holds genetic relevance*.* The risk of certain types of cancer appears to be modulated by *LRRK2-*PD mutations [3]; moreover, noncoding polymorphisms in the *LRRK2* locus are associated with an increased risk of leprosy [4] and inflammatory bowel disorder [5]. Of note, though *LRRK2* is an ubiquitous gene, its expression is elevated within the immune system, and plays an important role in glia [6], suggesting that tissue homeostasis in health and disease may be regulated by the immune system through LRRK2 activity. The protein product of the *LRRK2* gene is an enzyme (∼280 kDa) containing active kinase and GTPase catalytic sites surrounded by protein interaction domains [7–8]. Over the past 10 years, copious efforts have been dedicated to the dissection of the LRRK2 function and to the search for LRRK2 substrates and interactors.

## LRRK2: one protein — multiple partners — multiple functions

A wide variety of different functions have been associated with LRRK2 over the years, including various signaling pathways, autophagy, vesicle trafficking, vesicle recycling at the synapses, protein synthesis, gene expression, mitochondria homeostasis and immune system-specific activities [9]. A bioinformatics survey from our group revealed that 269 putative LRRK2 interactors were published in peer-reviewed journals, and this figure was scaled down to 62 interactors when filters were applied to control for data replication [10]. This scale and variety of putative LRRK2 interactors suggests that LRRK2 may behave as a ‘date-hub’, term that, in systems biology, refers to a dynamic protein capable of forming different complexes at different locations and times [11]. Under this perspective, LRRK2 may be capable of interacting with different proteins, therefore, controlling a plethora of diverse functions based on the complexes formed in different tissues, cell types, stages of development or under specific stimuli [12]. The date-hub hypothesis is intriguing because it provides a plausible reason as to why multiple functions and so many partners have been associated with LRRK2 over the past decade, and why the search for the unique LRRK2 role has revealed challenging. In fact, the concept of just one singular LRRK2 function may be misleading, and the experimental design (cell type, moment in development and experimental conditions) might play a determinant role in how the LRRK2 complex is formed, affecting the function that LRRK2 supports and that the experiment describes. Therefore, more precision is needed in characterizing the model system in which the study of LRRK2 is conducted. Another consequence is that among all the possible LRRK2 functions, only few will be relevant to disease. It will therefore be necessary to define a general group containing all LRRK2 functions among which the smaller subset of disease-associated ones can be identified.

## PD and the autophagy–endolysosomal pathway

**Figure 1.**
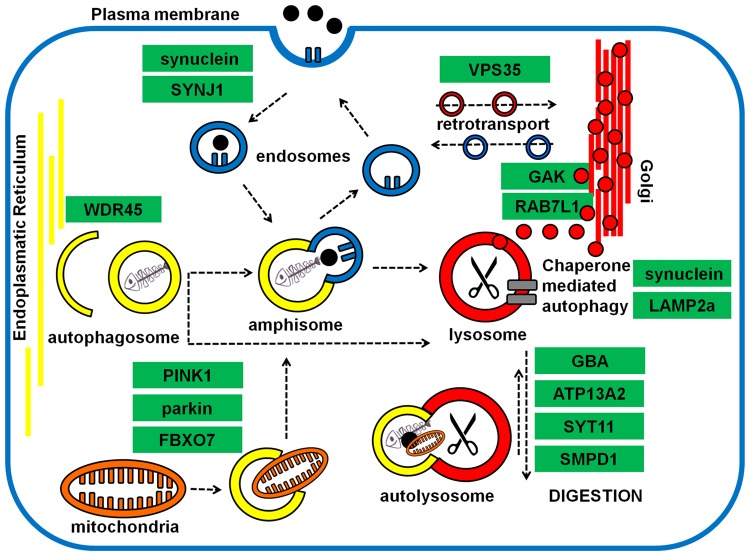
PD genes and autophagy-endolysosomal pathway(s). Parkinson's disease genes and their possible involvement in autophagy and endolysosomal pathway.

One of the functions LRRK2 has been associated with is autophagy, in the more general scenario of the endolysosomal system. This specific association is appealing because all the diseases associated with LRRK2 have been, to different extents, related with alterations of autophagy. In the case of PD, this association echoes results of functional studies performed with other PD genes (Figure 1) opening to the possibility that alterations in the autophagy–endolysosomal pathway might be an underlying common mechanism at the base of PD neurodegeneration.

*SNCA* was the first gene being associated with PD by either mutations [13] or copy number variations [14]; **α-synuclein**, the protein product of the *SNCA* gene, misfolds and deposits as amyloid material forming Lewy bodies, one of the pathological hallmarks of PD. The function of α-synuclein is still not known; it has been suggested that it may exert a chaperone activity at the synapse facilitating vesicle dynamics [15]. Mutated α-synuclein is known to impair chaperone-mediated autophagy (CMA) [16], and it is assumed that a change in the balance between its production and degradation rates may lead to its accumulation during PD. PD genome-wide association studies (PD-GWAS) suggested different candidate genes as risk factors for sporadic PD [17] among which **RAB7L1** and cyclin-G-associated kinase (**GAK**) were further proposed to interact with LRRK2 forming a complex to promote clearance of Golgi-derived vesicles through macroautophagy [18,19].

**VPS35** (vacuolar protein sorting-associated protein 35) is mutated in rare forms of familial PD; it is a component of the retromer complex that mediates the distribution of proteins in the endosome–Golgi–lysosome network. Mutations in VPS35 have been associated with alterations in the retromer complex with abnormal trafficking of the autophagy protein ATG9A leading to consequent autophagy impairment [20]. Additional studies showed that loss of (as well as mutations in) VPS35 causes an additional abnormal trafficking of the lysosomal protein **LAMP2a** (lysosome-associated membrane protein 2), involved in CMA with consequent impairment of α-synuclein degradation [21]. LAMP2a is another protein proposed in the PD-GWAS as a candidate risk factor for sporadic PD [17]; its levels were indeed found to be reduced in the *substantia nigra* and *amygdala* of PD cases at a level shown to be sufficient to impair CMA and increase the half-life of α-synuclein [22]. **WDR45** (WD repeat domain 45) is a protein mutated in neurodegeneration with brain iron accumulation presenting with Parkinsonism [23]. Only very few studies are available; however, WDR45 has been proposed to control autophagosome elongation [24]. **ATP13A2** (ATPase 13A2) is a lysosomal type 5 P-type ATPase mutated in the Kufor-Rakeb syndrome, a juvenile form of Parkinsonism presenting with dementia [25]. Alterations in ATP13A2 have been associated with lysosomal impairment, α-synuclein accumulation and a decrease in cathepsin D activity [26]. Recently, it has been proposed that ATP13A2 may be capable of regulating **SYT11** (synaptotagmin 11), another protein identified in PD-GWAS as a possible PD risk factor [18]. The ATP13A2/SYT11 interaction may control lysosomal functionality, autophagy pathways and α-synuclein clearance [27]. **GBA** (β-glucocerebrosidase) is an enzyme known for its association with Gaucher's disease, a lysosomal storage disorder caused by homozygous mutations reducing the hydrolytic activity of GBA with consequent built-up of undigested, highly toxic glucocerebroside within the lysosomes. Heterozygous mutations in GBA cause a 20-fold increase in the risk of PD [28]. Inhibition of autophagy by inactivation of protein phosphatase 2A (PPP2A) [29] as well as engulfment of the endoplasmic reticulum (ER) with misfolded GBA protein and consequent induction of ER stress [28] were postulated as a causal link between GBA mutations and PD. Recently, GBA mutations (and enzyme deficiency) were associated with a peculiar, final stage of macroautophagy where lysosomes are recycled following the engulfment and digestion of the autophagosomes [30]. Mutations in **PINK1**, **parkin** and **FBXO7** (F-box only protein 7) lead to early-onset PD; the relevance of these proteins in mitochondria quality control and mitophagy is well established [31]. **SYNJ1** (synaptojanin 1) is mutated in rare forms of PD [32]. Although not well studied to date, loss of SYNJ1 causes abnormal endolysosomal trafficking with accumulation of late endosomes and autophagosomes in cone photoreceptors of zebrafish with concomitant defects in autophagosome maturation [33]. Finally, a mutation in **SMPD1** (sphingomyelin phosphodiesterase-1), a protein known for its association with Niemann-Pick lysosomal storage disease, was recognized as strong risk factor for PD [34]. SMPD1 is a type of sphingomyelin phosphodiesterase relevant for lysosomal homeostasis [35] and, potentially, autophagy [36].

The alluring scenario of a common pathogenic mechanism connecting all the genes related with PD was further emphasized by the fact that autophagy-deficient mouse models (following Atg7 knockdown) are affected by presynaptic accumulation of α-synuclein, thus recapitulating one of the hallmarks of PD pathogenesis [37].

All this evidence, paired with the fact that macroautophagy constitutes a reasonable therapeutic target with different drugs already approved, has led autophagy and the endolysosomal pathway, among other LRRK2 functions, to gain momentum, becoming the center of attention of many laboratories worldwide.

## LRRK2 and the autophagy–endolysosomal pathway

**Figure 2.**
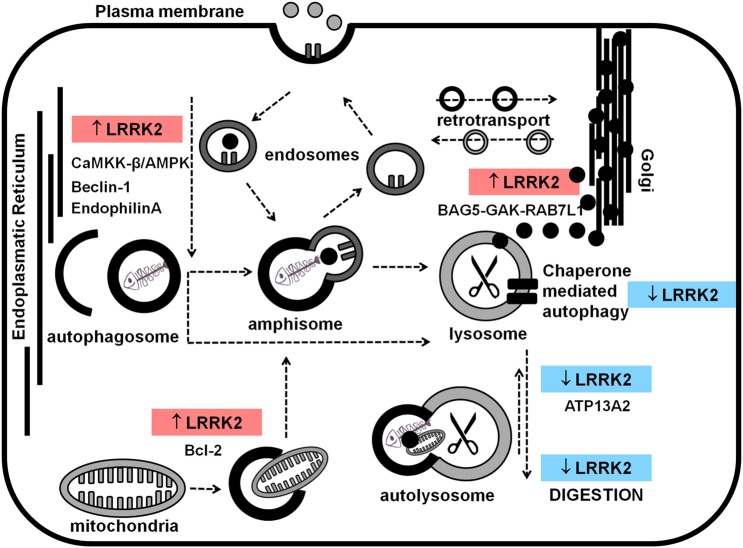
LRRK2 and autophagy-endolysosomal pathway(s). LRRK2 and its possible involvement in autophagy and endolysosomal pathway (red boxes = increase, blue boxes = decrease in, autophagy).

The first reports suggesting a role for LRRK2 in autophagy showed, in cellular models, that overexpression of G2019S-LRRK2 in SHSY5Y cells was sufficient to induce neurite shortening with a mechanism dependent on macroautophagy [38], whereas LRRK2 silencing triggered an increase in the basal macroautophagy flux [39]. These first two works, published between 2008 and 2009, set the stage for many studies that investigated the intriguing, though still incomplete and sometimes controversial, association of LRRK2 with the autophagy–endolysosomal pathway (Figure 2).

LRRK2 has been studied in different model systems through overexpression (both wild-type and mutant sequences), knockdown/knockout, chemical inhibition of kinase activity (while increase in kinase activity can normally be achieved by expression of the G2019S-LRRK2 sequence), and in patient-derived cell cultures. As detailed below, the investigation of LRRK2 modulation over macroautophagy has produced a complex and controversial set of results (Table 1) showing (i) both a positive and a negative regulation of LRRK2 over macroautophagy; (ii) a role for LRRK2 in both the initiation and the final phases of autophagy and (iii) similar outcomes following LRRK2 kinase activity inhibition (achieved by chemical inhibition and/or LRRK2 knockdown/knockout) or increase (achieved by overexpression of G2019S-LRRK2).

**Table 1:** Open challenges for the study of LRRK2 in the context of autophagy-endolysosomal pathway(s)

**Discordant reports regarding the role of LRRK2 in macroautophagy**	**Positive** and **negative** regulation of LRRK2 over macroautophagy
	LRRK2 modulation of the **initiation** and **termination** phases of macroautophagy
	Similar outcomes following both **inhibition** and **increase** of LRRK2 kinase activity
**Technical challenges when integrating reports concerning the role of LRRK2 in macroautophagy**	Wide variety of **model systems** (e.g. different cell lines, human vs. animal cells and different types of primary cultures)
	Experiments performed at different LRRK2 **expression levels** (i.e. overexpression vs. endogenous level)
	Outcomes achieved by studying **mutant** LRRK2 are difficult to recapitulate with results obtained with the **wild-type** sequence
	Results obtained with mutant LRRK2 are difficult to summarize; there are too many different mutations (G2019S appears to be the most frequently studied), results are inconsistent showing G2019S-LRRK2 to both potentiate and repress autophagy depending on the model system/experimental procedure.
	**Chemical inhibition** of LRRK2 kinase activity is difficult to compare with **knockdown/knockout** strategies where the entire LRRK2 protein is removed (comprising the kinase, GTPase and protein interaction domains)
	Unavailability of drugs and techniques to dissect **LRRK2 kinase vs. GTPase** activities
	A wide variety of strategies to stimulate/repress macroautophagy coupled with different model systems may result in **different macroautophagy pathways** and/or control feedback loops to be activated

As first, overexpression of LRRK2 suggested a possible involvement in a dual control over macroautophagy and lysosomal functionality by activating macroautophagy through a calcium signaling cascade controlled by the calcium-dependent protein kinase kinase-β (CaMKK-β)/adenosine monophosphate (AMP)-activated protein kinase (AMPK) pathway, and by reducing degradation through a concomitant increase in lysosomal pH [40]. Along the same route, expression of mutant LRRK2 (R1441C, Y1699C and G2019S-LRRK2) in astrocytes resulted in formation of enlarged, but nonfunctional, lysosomes, with altered pH and increased expression of ATP13A2 (in the case of G2019S-LRRK2) [41]. As these defects were rescued by chemical inhibition of LRRK2, the increase in kinase activity following LRRK2 mutation may be responsible for the observed lysosomal alteration. Chemical inhibition of LRRK2 kinase activity confirmed a role for LRRK2 in the modulation of the macroautophagy flux, with apparently discordant results suggesting (i) a positive, mTOR-independent–Beclin-1-dependent [42,43] and (ii) a negative [44] regulation of macroautophagy. Knockdown of LRRK2 (i.e. silencing of its kinase activity) in immune cells resulted in impairment of macroautophagy and reduction of its degradative capacity [45]. However, a similar outcome was obtained in the opposite scenario through overexpression of G2019S-LRRK2 (i.e. increased LRRK2 kinase activity) with disruption of aggregosome formation and impairment in clearance when macroautophagy was assessed concomitantly with proteasome inhibition [46].

Other apparently conflicting results were obtained studying PD patient-derived cells carrying LRRK2 mutations. Studies in mutant fibroblasts suggested both increased based level of macroautophagy through activation of the MEK/ERK pathway (only for G2019S-LRRK2) [47] and reduced response to induction of macroautophagy following nutrient starvation and mTOR inactivation (for R1441G, Y1699C and G2019S-LRRK2) [48]. Neurons derived from PD fibroblasts carrying G2019S-LRRK2 revealed alterations in macroautophagy, however, at the level of autophagosome degradation [49].

LRRK2 animal models did not help in clarifying this complicated landscape. A first LRRK2 knockout mouse model showed a biphasic, age-dependent alteration in macroautophagy in the kidneys [50], whereas transgenic mice expressing G2019S-LRRK2 showed age-dependent degeneration of nigrostriatal pathway and dopaminergic neurons with autophagy and mitochondrial abnormalities [51]. Alongside macroautophagy, CMA has been analyzed as this process was hypothesized to be important for LRRK2 turnover. CMA was found to be impaired by mutant forms of LRRK2 mirroring a mechanism already suggested in the case of mutant α-synuclein [52].

Although the relevance of LRRK2 in the control of autophagy is well documented and accepted by the scientific community, it is clear that we are still missing many mechanistic details while many studies report controversial results in regard to the actual molecular mechanism through which this control is exerted. There may be multiple technical explanations for these inconsistencies (Table 1).

However, in the light of these sometimes confusing data, it may be worth considering again the dynamic ability of LRRK2 to form complexes as it has been previously discussed. In fact, the data-hub hypothesis may not only be relevant to the general array of LRRK2 functions; it may as well describe the role of LRRK2 in the context of autophagy. Different cell types and different triggering circumstances may cause LRRK2 to interact with different partners within the autophagy machinery enabling LRRK2 to control autophagy in slightly different ways. In fact, specific roles for LRRK2 in the control of macroautophagy have been described when LRRK2 was part of specific protein complexes. Particularly, the LRRK2-mediated control of mitochondria homeostasis through macroautophagy was described following interaction with the B-cell lymphoma 2 (Bcl-2) protein [53], and this observation was further corroborated by the finding that Bcl-x_L_ prevented macroautophagy alterations induced by chemical inhibition of LRRK2 kinase activity [54]. LRRK2 promotion of Golgi-derived vesicle clearance by macroautophagy was described only when LRRK2 was in complex with Bcl-2-associated athanogene domain cochaperone 5 (BAG5), GAK and RAB7L1 [18]. Specifically at the presynaptic terminal, LRRK2 was shown to be capable of regulating macroautophagy by phosphorylating EndophilinA, which in turn is capable of modulating the membrane curvature, thus controlling the recruitment of the autophagy machinery to the nascent autophagosome [55].

## Conclusion

LRRK2 has been associated with many different functions among which is autophagy. Autophagy has a clear role and impact in LRRK2-associated disorders. Since other PD genes have independently been linked to the autophagy–endolysosomal pathway, this might represent a common mechanism to PD neurodegeneration. However, even if the study of LRRK2 in the context of autophagy has been extensive, the complete picture is still unclear due to controversial results that are very difficult to recapitulate in an exhaustive, unifying hypothesis. I here suggest that, in addition to technical issues, one reason for this plethora of apparently discordant findings can be the specific complex forming behavior of LRRK2. This suggests that, in different experimental systems and cellular models, specific LRRK2 complexes may be formed and not necessarily be involved in the same control/function. Therefore, to understand the dynamic lynchpin activity of LRRK2, we should start shifting the focus of our attention from close-up (i.e. LRRK2 in isolation) to the wider perspective of the experimental context.

## Abbreviations

ATP13A2: ATPase 13A2;
Bcl-2: B-cell lymphoma 2;
CaMKK: calcium-dependent protein kinase kinase-β;
CMA: chaperone-mediated autophagy;
ER: endoplasmic reticulum;
ERK: extracellular signal-regulated kinase;
GAK: cyclin-G-associated kinase;
GBA: β-glucocerebrosidase;
LAMP2a: lysosome-associated membrane protein 2;
LRRK2: leucine-rich repeat kinase 2;
mTOR: mammalian target of rapamycin;
PD: Parkinson's disease;
PD-GWAS: PD genome-wide association studies;
SMPD1: sphingomyelin phosphodiesterase-1;
SYNJ1: synaptojanin 1;
SYT11: synaptotagmin 11;
VPS35: vacuolar protein sorting-associated protein 35;
WDR45: WD repeat domain 45.
